# Impact of Nitrogen Foamed Stimulation Fluids Stabilized by Nanoadditives on Reservoir Rocks of Hydrocarbon Deposits

**DOI:** 10.3390/nano9050766

**Published:** 2019-05-18

**Authors:** Klaudia Wilk, Piotr Kasza, Krzysztof Labus

**Affiliations:** 1 Department of Production Stimulation, Oil and Gas Institute—National Research Institute; Lubicz 25A Str., 31-503 Krakow, Poland; kasza@inig.pl; 2 Faculty of Mining and Geology, Silesian University of Technology, Akademicka 2 Str., 44-100 Gliwice, Poland; krzysztof.labus@polsl.pl

**Keywords:** nanoaditives, nitrogen foamed stimulation fluids, reservoir stimulation, rheology, formation damage, SEM

## Abstract

The first objective of this experiment was to improve the stabilization of N_2_ based foam with nanoparticles as an alternative to typical fracturing fluid, which consists of a gelling agent (HPG—hydroxypropyl guar). The second objective of the project was to investigate the damage caused by nanoparticle–based nitrogen foamed fracturing fluids (F.F) on a reference sandstone, using permeability and porosity tests, optical microscope with a Profilometer, and a scanning electron microscope (SEM). The properties of F.F with two types of SiO_2_ nanoparticles (hydrophilic fumed silica Areosil 300 and silica sol U-2 obtained by the sol-gel method), such as rheology and core damage, were investigated. The discussion of this research results is based on the stability tests carried out with the use of rheology and the foam half-life, formation damage ratio, and observation of exposed samples using SEM and the Profilometer. The permeability and porosity damage ratios of the damaged core samples were found to decrease when nitrogen foamed fluids were used. These results were confirmed with the Profilometer and SEM images. The experimental data showed that the foam stability increased when silica (SiO_2_) nanoparticles were added. SiO_2_ nanoparticle-surfactant-stabilized foam for fracturing is superior to traditional water-based fracturing fluids and causes lower core permeability damage than a traditional F.F.

## 1. Introduction

One of problems facing the oil industry lies in achieving the production of maximum amounts of oil remaining in reservoirs after natural energy conditions have been used. In many hydrocarbon reservoirs that are exploited worldwide, the mining has approached the final phase [1]. By means of the first extraction methods utilizing the reservoir energy, it is possible to obtain only approximately 5% to 20% of resources [2,3]. Therefore, for many oil companies, the development of hydrocarbon stimulation methods is a priority. Stimulation treatments, such as HF [4,5,6], matrix acidizing, acid fracturing [7,8,9], or EOR [6,10,11,12] are common techniques used to increase the extraction productivity. In all aforementioned cases, the stimulation (injection) fluid is a crucial element and must meet special requirements depending on the goal of the application [13,14,15]. After stimulation treatment, a part of this fluid remains within fractures, causing damage to the formation and reducing the stimulation effectiveness [16,17,18]. Therefore, attention has been drawn to the possibility of the application of fluids energized with gases with the addition of nanoparticles, thereby reducing the water content in the injection fluid and also increasing the stabilization of the process fluid during stimulation treatments [19,20,21,22,23]. The advantage of energized fluids consists also in the increased fluid recovery after fracturing due to the natural energy of the gaseous fluid component [24,25]. Because gas decompression occurs during pressure reduction and fluid reception after treatment, the dissolved gas helps to recover the pumped fluids and facilitates well cleaning [26,27]. Moreover, the high viscosity of the foam allows better transport of the proppant and more effective placement of it in the fracture without excessive decreases of the proppant material [28,29]. It also ensures good control of the fluid filtration to the rock matrix and to natural fractures during fracturing. Their application allows the amount of water necessary for treatments to be significantly reduced [16,30,31], limiting the possibility of clay minerals swelling in the deposit, thus causing reduced permeability [32,33]. In this case, when the fracturing fluids are water-based, so-called permeability damage can occur, caused by the swelling of clay minerals or the action of other physical and chemical mechanisms proceeding in the fractured formation [34]. This reduces the reservoir rocks’ permeability at the stage of drilling, hydraulic fracturing, production, and other reservoir operations, resulting in decreased reservoir productivity [35], which translates directly into economic effectiveness.

Nanotechnology is a fast developing field, offering a multitude of potential applications and benefits [36,37,38,39,40,41]. Nanoparticles feature a number of advantages during reservoir stimulation with foamed fluids, such as: The ability to increase foam stability [42]; stabilization of small bubbles, which increases the viscosity—which is necessary for the effective transfer of the proppant material [43]; smaller size when compared to rock fractures and pores [44], which allows for more effective transport to the surface of the post-treatment fluid during the process of well cleaning; ability to reduce the migration of solid particles [45]; environment-friendly [46]; ability to reduce corrosion. More importantly, the mechanism of nanoparticles’ movement and action [47,48,49,50], and also of foam stabilization by nanoparticles, differs and is more effective than that utilizing surfactants and emulsifiers. After the stage of pumping and placement of the proppant in the fracture, foam loses its stability and viscosity, and foam bubbles regenerate during fluid recovery after treatment [51]. Post-hydraulic fracturing cleanup of the well and the largest possible flowback become very significant factors regarding nanoparticles with strong penetration abilities. After the use of a nitrogen foam stabilized with silica, to achieve consolidation, collection, and elimination of the nanoparticles to prevent negative consequences for the environment, the magnetic method can be used to collect nanoparticles. Some remedial methods exist, among others, such as methods creating a composite nanoparticle of SiO_2_ and hexaferrite. The authors of [52,53,54,55] propose that these materials are chemically stable in air at operating temperatures. The use of permanent magnet separators ensures that nanoparticles can be disposed, thus protecting the environment after hydraulic fracturing treatment.

## 2. Materials and Methods

Using tap water as the base, a foamed stimulation fluid was formed by the addition of N_2_, a foamer, nanoparticles, and natural polymer. Silicon dioxide (U-2) from Industrial Chemistry Research Institute, Warsaw, Poland, in the form of a 23% water solution, was the first type of used nanoparticle. The silica sol was obtained by the sol-gel method. Tetraethoxysilane (TEOS) was a direct substrate used to obtain the silica sol. The reaction was carried out in a water-alcohol medium in the presence of ammonia solution within a pH range of 10.97 to 11.00. The process proceeded as follows: Anhydrous ethanol, ammonia solution, and distilled water were mixed in an Erlenmayer flask using a mechanical mixer. The pH of the formed solution was measured after 15 min. The pH value of reaction mixture prepared during the process of silica sol formation was strictly controlled to ensure repeatability of the SiO_2_ particle size. Then, TEOS was added while ensuring continuous mixing. In the initial stage of synthesis, the reaction mixture (sol) was clear; after a dozen or a few dozen minutes, solution opalescence was observed. The process was stopped after 3 hours of intensive mixing. Based on the photon correlation spectroscopy, the sol particle size was found to be 30 nm. To obtain a 23% SiO_2_ solution, the obtained silica sol was concentrated through the evaporation of solvents to a defined volume.

Silica nanoparticles, Areosil 300 was the second type of used nanoparticle, and was obtained from Evonik Industries AG, Essen, Germany. The colloidal silica, referred to as ‘fumed silica’, because it is produced through continuous flame hydrolysis, was formed via combustion of silica tetracholoride SiCl_4_ in an oxygen-rich flame. The silica powder features an extremely low density of 90 g/L and a high specific surface area of 300 m^2^/g (+/− 30 m^2^/g). Areosil 300 is a mixture of lipophobic and hydrophilic nanoparticles (LHP) with a mean particle size of approximtely 7 nm. Its composition contains silicon dioxide (SiO_2_) > 99.8%, aluminium oxide (Al_2_O_3_) < 0.05%, titanium dioxide (TiO_2_) < 0.03%, hydrogen chloride HCl < 0.025%, and iron III oxide (Fe_2_O_3_) < 0.003%. pH ranged between 3.70 and 4.70. Initially, nanoparticles in the form of a powder (AEROSIL 300) or of a suspension (U-2) were added to the tap water at room temperature, then the solution was stirred with a mechanical mixer for 4 to 5 min. After that period, the sample was subject to ultrasonic wave action using a homogeniser for 4 min, at an amplitude of 70%. Anionic foaming agent A from CESI Chemicals, Houston, TX, USA was added next (4 mL/L), and finally, optionally polymer W (natural, fast hydrating guar gum for oil field applications) (made by Weatherford) was added at an amount of 1 g/L. Agents A and W were used based on our previous work to assess the best additives for foamed fluids [30,56]. Samples of model rock material, taken from a depth of approximately 300 to 400 m, originating from a deposit situated in the upper part of the Lower Istebna beds, were taken for laboratory tests to determine the degree of damage. These strata exist mainly in the form of thick-banked massive fine- and medium-grained sandstones with clayey-limy binder with subordinate shale banks. These strata exist between shaly sediments, mainly in the form of thick-banked massive fine- and medium-grained sandstones with clayey-limy binder. 

### 2.1. Viscosity of the Stimulation Fluids

**Figure 1:**
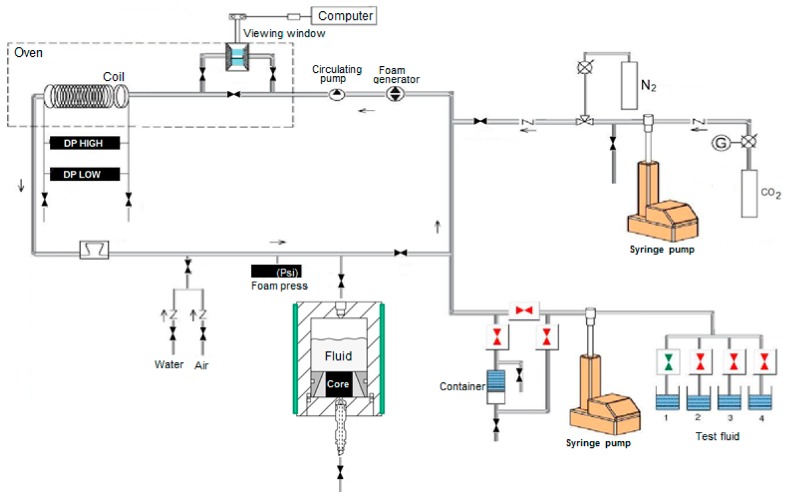
Measuring system to study the damage to the core by fracturing fluids with addition of N_2_/CO_2_ gases.

To prepare fracturing fluids with the addition of nanoparticles to carry out rheological measurements, the procedure described in Section 2 was followed. The fluid was then introduced to the tubes of a pipe rheometer designed specifically to measure the rheological properties of foamed systems under extended pressure and temperature conditions and stirred at a rate of 350 s^−1^. To study the rheological properties of the foamed fluids, the base fluid was first foamed with nitrogen. To this end, approximately 500 mL of the tested fluid was placed in the fluid container (Figure 1). Then, by means of pumps, it was pumped into the tubes of the measuring system. After filling and venting, fluid circulation was started in the measuring system, stabilizing at the same time the temperature and pressure (6.89 MPa, T = 23 °C). Next, gas was additionally pumped to the measuring system, circulating the fluid continuously at a shear rate of 350 s^−1^. At the same time, the fluid was partially collected from the system, and then a partially foamed fluid, thereby increasing the gas share in the foam. The process was carried out till the moment that 50% or 70% of the foam quality was obtained, which was controlled by a densimeter. Once the foam quality stabilized, measurements of the rheological properties were started in accordance with the prepared test plan. The stability test lasted 80 min, at a pressure of 1000 psi, maintaining a shear rate of 100 s^−1^. To measure the rheological properties during measurement loops (at minute 13, 25, and 38), the shear rates were assumed as follows: 40, 100, 200, 300, 200, 100, and 40 s^−1^. During a measurement loop, the shear rate was kept at each of the aforementioned levels for 60 s to obtain a stable result. Between measurements, the foam was stirred at a rate of 100 s^−1^ during 10 min (Table 1, Table 2 and Table 3). The foam half-time was determined after generating foam of a 50% or 70% quality; the fluid flow through the rheometer was stopped and the foam was closed in the measurement chamber to maintain static measurement conditions. This was defined as the time after which half of the water phase was separated from the generated foam [57], and it is an important parameter used to describe foam stability. Table 4 presents results of the half-time measurements for S.F.

### 2.2. Induced Formation Damage

To a large extent, the damaging tests consisted in pumping appropriate fracturing fluids through the cores, causing damage to the core material at the assumed pressure difference, which is the case during actual reservoir stimulating treatments.

To simulate the formation damage by fracturing fluids, taking into account the impact of process fluids on the reservoir rock, a measuring system to test the damage to the cores was used. To identify the reservoir formation damage, it was necessary to appropriately prepare the cores. Samples were prepared from the core material to perform tests of the rock damage by a fracturing fluid (non-foamed or with a 50% content of N_2_). First, core plugs were cut out by a diamond crown, 3.81 cm in diameter and approximately 2.54 cm high. After cutting, they were dried and placed in a desiccator. A decision was made to cut plugs of a larger diameter to have the maximum pore volume and front surface of the core possible during the test, on which the filtration cake formed. Core plugs prepared in such a way were subject to measurements of the permeability coefficient for gas and of the porosity ratio. The results the measurements are specified in Table 5. Then, the core plug was set in the measuring chamber using high-temperature silicone. Next, the remaining components of the measuring chamber were screwed together and it was left for approximately 24 h. After that period, the chamber was thermostated up to 60 °C and the measurement was started. The core was initially saturated with a 2% KCl solution at a constant rate by means of a constatimetric pump and then the chamber was filled with an appropriate fracturing fluid, and a pressure of 6.89 MPa (1000 psi) was applied. After opening a valve at the chamber bottom, the core damaging started, lasting 50 min. in total.

### 2.3. Rock Cores Sample Damage Examination

The use of an HRM-300 3D (HRM-300 Series, Huvitz, Dongan-gu, South Korea) optical microscope with a profiler and digital equipment and Panasis software allowed the rock samples’ damage to be imaged. For each core, after damage, 3 surface profiles were made using a reference plane—the surface without contact with the stimulation fluid (without filtration cake). The determined profile was comprised of the area from the core center to the wall of the rock mini-cylinder (5000 µm). The cake height was determined taking into consideration the average roughness from the roughness profiles along selected measurement sections.

FEI Quanta 650 FEG (Thermo Fisher Scientific, Hillsboro, OR, USA) scanning electron microscope was used to obtain pictures and SEM analyses. The Quanta microscope was equipped with a field emission gun (FEG). The core photographs were made using a detector of backscattered electrons (BSE). Based on differences in the gray image scale, a phase contrast was visible on the sample surface (heavier minerals are lighter on the image, while lighter ones are darker). A high and low vacuum was used for imaging. A low vacuum was used to avoid ‘sample charging’ (charge gathering in non-conducting places). The degree of damage was compared for cores, through which non-foamed fracturing fluid was pumped, with cores through which foamed fracturing fluid was pumped.

To observe the core plug damage not only on the front surface, but also outside, the core was split transversally into two parts, reproducing a natural rock fracture. It enabled more detailed observations of the range of the rock sample damage by fracturing fluids.

## 3. Results

### 3.1. Viscosity Measurements

**Figure 2:**
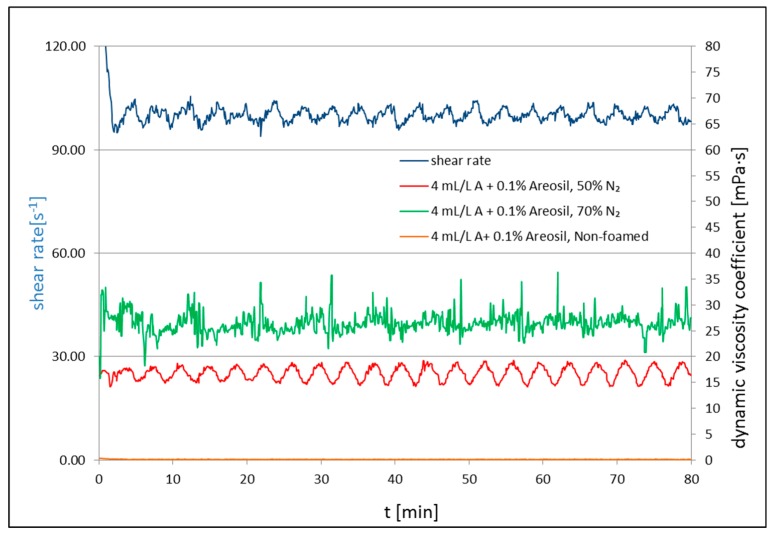
Viscosity of non-foamed and N_2_ foamed fluid of 50% and 70% quality at 23 °C at a shear rate of 100 s^−1^.

**Figure 3:**
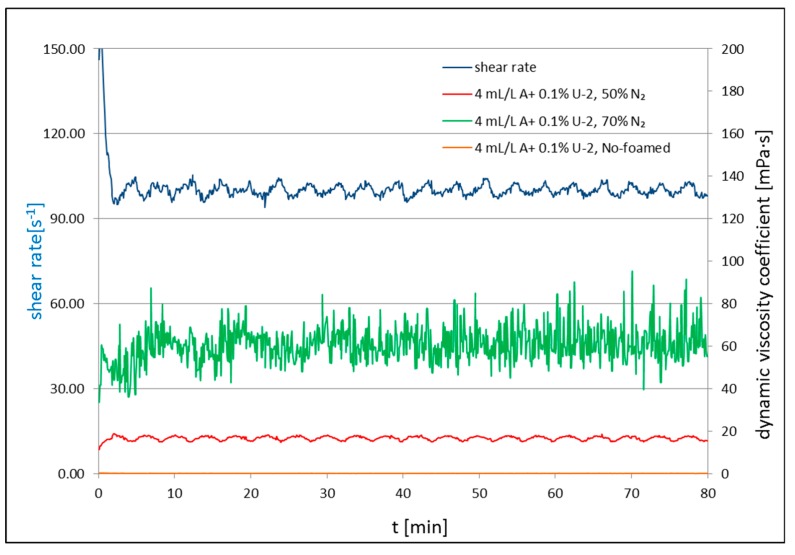
Viscosity of non-foamed and N_2_ foamed fluid of 50% and 70% quality at 23 °C at a shear rate of 100 s^−1^.

**Figure 4:**
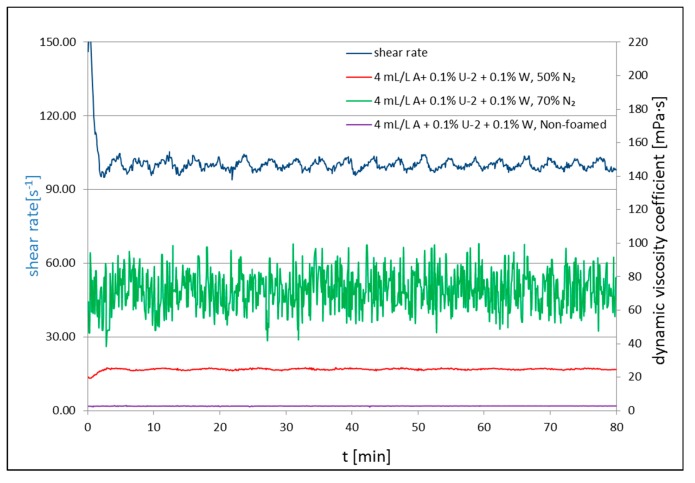
Viscosity of non-foamed and N_2_ foamed fluid of 50% and 70% quality at 23 °C at a shear rate of 100 s^−1^.

Figure 2, Figure 3 and Figure 4 present the results of the rheological property measurements for non-foamed and with nitrogen addition fracturing fluids. Measurements of the rheological properties for all tested foamed and non-foamed fluids were carried out at 23 °C. The rheological parameters (*n*′ and *K*′) are presented in Table 1, Table 2 and Table 3, where *n*′ is the dimensionless flow index and *K*′ is the consistency factor.

Figure 2, Figure 3 and Figure 4 present the apparent viscosity registered during the test for the processed fluid solutions with the addition of a surfactant, nanoadditives Aerosil 300 (Figure 2) or U-2 (Figure 3), and polymer in certain cases (Figure 4). For each composition of additives, two tests were performed: The measurement of *n*′ and *K*′ (Table 1, Table 2 and Table 3) and the measurement of the apparent viscosity over time (Figure 2, Figure 3 and Figure 4). Each time, basic rheological parameters were tested for foam of the 50% and 70% quality. The nanoadditive, Aerosil 300, was used in the first series of tests. The initial viscosity of 50% foam with the addition of only a foamer and the nanoadditive was 16 cP and 26 cP (*Q_f_* = 70%) at 100 s^−1^. The non-foamed fluid featured a viscosity of approximately 2 cP at 100^−1^. In the second series of tests, the U-2 nanoadditive was used at the amount of 0.1% vol. The viscosity with the addition of only a surfactant and the nanoadditive was 15 cP in the case of foam at a temperature of 23 °C and 50% quality, and 52 cP for the foam of the 70% quality. After adding 0.1 wt.% of natural polymer to U-2 nanoparticles, the viscosity went up to 22 and 55 cP for the tested foam qualities, respectively. The nanoparticle addition increased the stability of the foamed fluid, which was confirmed by the authors of [58]. The increased stability was also confirmed by analyzing the half-time. It increased 12-fold in the case of the 50% nitrogen content in the fluid with U-2 addition and polymer, and 6.5-fold for the 70% foam as compared with the fluid without the SiO_2_ addition (Table 4).

### 3.2. Formation Damage Evaluation

**Figure 5:**
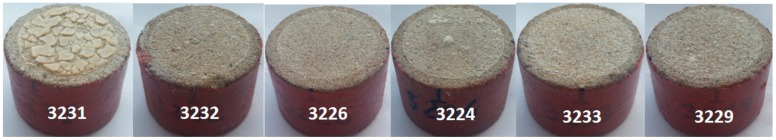
The cores surface after damage with S.F.

The permeability coefficient was significantly decreased, in particular in the case of cores treated with non-foamed process fluids. Foamed fluids caused a smaller permeability and porosity reduction than non-foamed fluids. The biggest damage to permeability was caused by non-foamed fluids with the addition of polymer W (Figure 5). The estimated permeability damage was approximately 20% smaller for foamed fluids as compared with fluids without the nitrogen addition (Table 5). The concentration of the nanoparticle suspension, well-dispersion solution, injection rate, and pore volume injected are the most important parameters affecting the permeability impairment [59].

**Figure 6:**
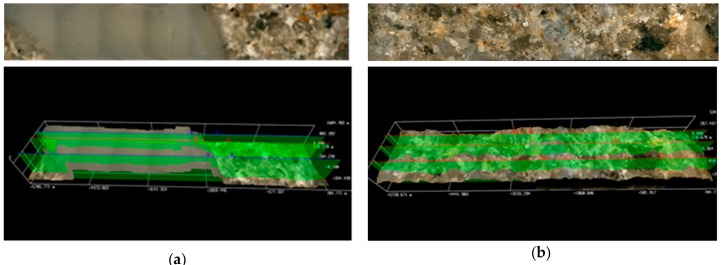
Results of microscopic analysis of the front surface of core No 3231 and 3232 after the damaging test S.F.: (**a**) 1a, (**b**) 1b.

**Figure 7:**
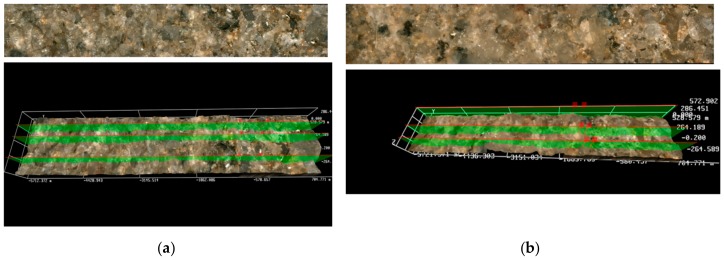
Results of microscopic analysis of the front surface of core No 3226 and 3224 after the damaging test S.F.: (**a**) 2a, (**b**) 2b.

**Figure 8:**
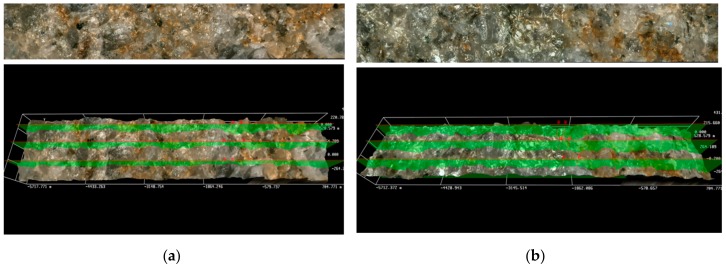
Results of microscopic analysis of the front surface of core No 3233 and 3229 after the damaging test S.F.: (**a**) 3a, (**b**) 3b.

The filtration cake height was determined by the 3D software in the optical microscope, using an arithmetical mean of three selected areas on the front surface of the tested rock sample. The average height of the cake for non-foamed fluids ranged between 1161 and approximately 108 µm. Instead, in the case of cores treated with foamed fracturing fluids, the measured filtration cake was definitely thinner and was from a few dozen to approximately a dozen µm thick. Figure 6 presents the front surface of cores 3231 and 3232 after pumping through the Aerosil addition, non-foamed (Figure 6a) and foamed (Figure 6b), respectively. A layer of filtration cake is especially visible on the profile of the non-foamed fluid (Figure 6a). Results of the presented tests show that the N_2_ foamed fluid based on nanoparticles with the addition of a foamer and U-2 additive is least invasive (Figure 7b). Only small traces of a filtration cake in the form of an uneven coating are visible on the surface. In the case of the filtration of fluid based on polymer with nanoparticle addition, the filtration cake is best visible (Figure 8a,b). Its thickness in the case of the U-2 application in a non-foamed fluid is estimated at approximately 170 µm (Figure 8a), while in the case of foamed fluids, at approximately 110 µm (Figure 8b).

**Figure 9:**
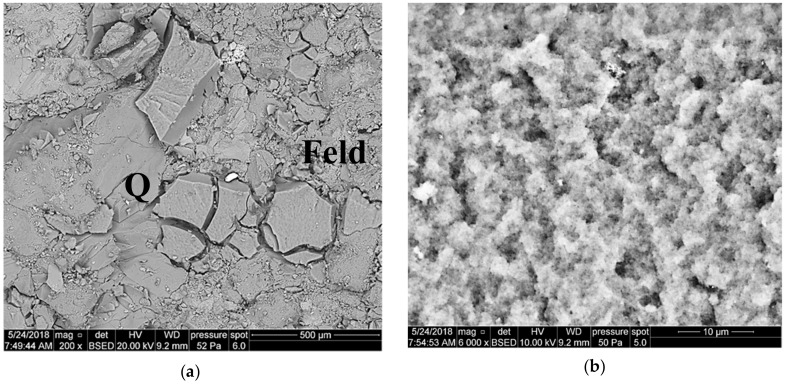
SEM image of the 3231 core face after contact with Areosil S.F. 1a, (**a**) top view of the core face; Q—quartz, Feld—feldspar, (**b**) top view of the core face at a high magnification.

Figure 9 presents the front surface of core 3231 after the core damage with the fluid with Aerosil additive—1a. The filtration cake coating (Figure 9a) is a silica gel; it exists only in fragments, is strongly crushed, and fills cavities between detrital rock components (quartz and feldspars). It is possible to distinguish one type of cake fragments: Fragments with a flat, but slightly lumpy surface. Figure 9b shows a filtration cake coating (silica gel) at high magnification. The surface is uneven, and relief elements are spread irregularly. The cake structure is not uniform, and it seems to be formed of grains much smaller than 1 μm.

**Figure 10:**
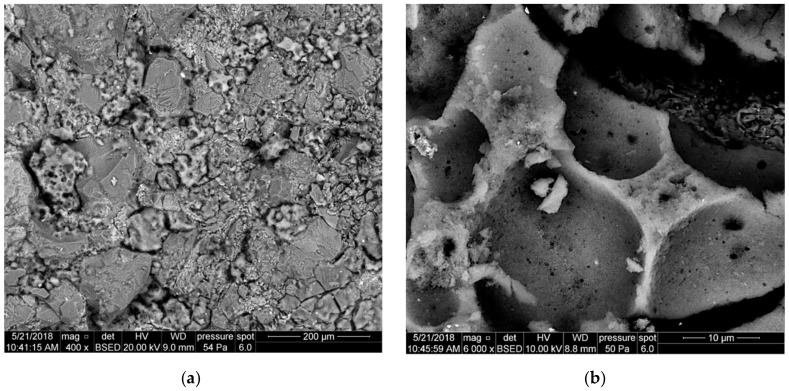
SEM image of the 3232 core face after contact with foamed Areosil S.F. 1b, (**a**) top view of the core face, (**b**) top view of the core face at a high magnification.

In the case of the foamed fluid application—1b—the front surface of the sample is covered with a highly crushed coating, filling cavities between the quartz grains (Figure 10a). In the close-up, one can see fragments of the cake with a porous surface, with finer cavities up to a dozen or so micrometers in diameter after gas bubbles (Figure 10a). The structure reveals the sub-micron elements forming the coating.

**Figure 11:**
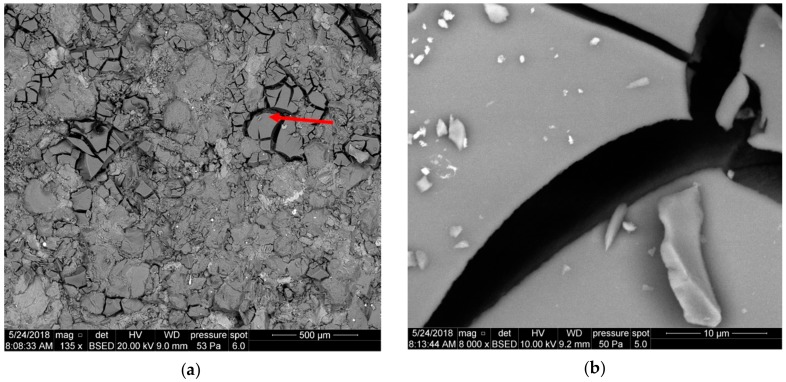
SEM image of the 3226 core face after contact with U-2, S.F. 2a, (**a**) top view of the core face, (**b**) top view of the core face at a high magnification.

Figure 11 presents the surface of sample 3226. The cake coating is strongly crushed and fills cavities between detrital rock components (Figure 11a). It is possible to distinguish polymer fragments with a smooth surface. Fractures are visible on the magnification of the cake fragment; the small white crystals are KCl, which crystallized from the pad fluid (Figure 11b).

**Figure 12:**
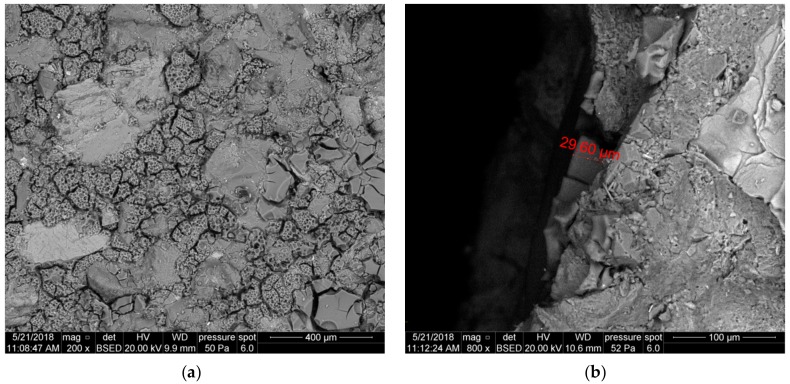
SEM image of the 3224 core face after contact with foamed U-2 S.F. 2b, (**a**) top view of the core face, (**b**) side view of the core face.

Figure 12a shows a polymer coating, which is strongly crushed and fills cavities between the detrital rock components (quartz and feldspar) of sample 3224. It is possible to distinguish two types of polymer fragments: (1)—Fragments with a smooth surface, with noticeable cavities after gas bubbles, a few dozen μm in diameter; (2)—fragments with a porous surface, covered with finer cavities after gas bubbles, up to a dozen or so μm in diameter. The presence of those two types suggests zonal differentiation of the fluid viscosity and surface tension. Figure 12b presents the front surface of sample—a side view. The cake coating, approximately 30 μm thick, is visible only on the surface.

**Figure 13:**
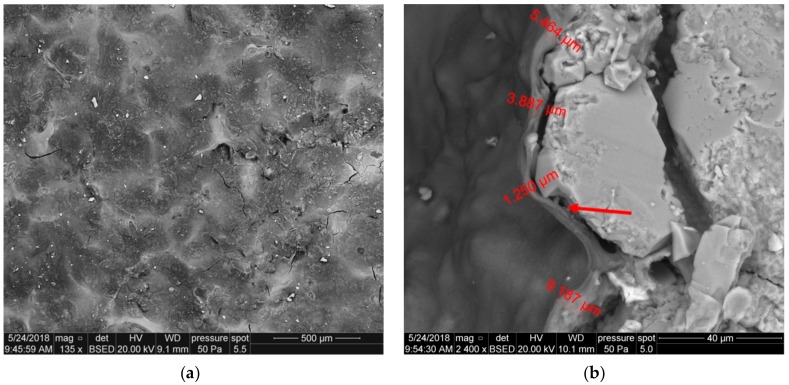
SEM image of the 3233 core face after contact with U-2 S.F. 3a, (**a**) top view of the core face, (**b**) side view of the core face.

The filtration cake coating on core 3233 is characterized by considerable continuity, which is related to the addition of polymer W, but with a finely diversified relief: Shallow pseudo-polygonal cavities and a few irregular fractures are marked. Occasionally, existing small mineral fragments are dispersed on the polymer surface, as shown in Figure 13a. Figure 13b presents the front surface of sample 3233—a side view. A uniform polymer coating (red arrow) is a few μm thick.

**Figure 14:**
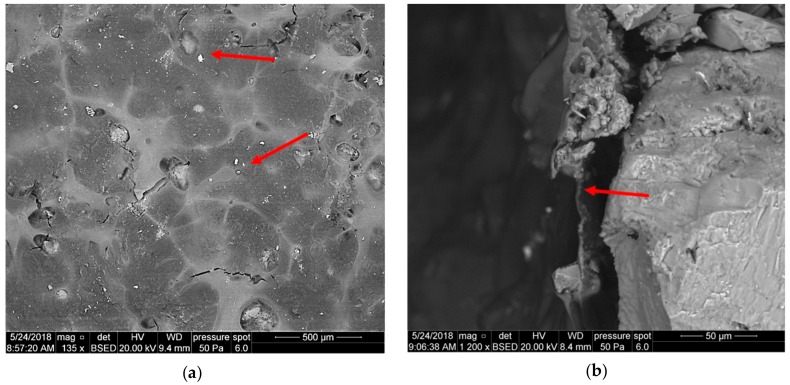
SEM image of the 3229 core face after contact with foamed U-2 and polimer S.F. 3b, (**a**) top view of the core face, (**b**) side view of the core face.

In the case of the foamed fluid showed in Figure 13b, the coating on the core surface (Figure 14a) is also continuous, with a finely diversified relief and shallow pseudo-polygonal cavities. Contrary to sample 3223 (Figure 13a), oval cavities are visible, probably related to gas bubbles, with dimensions up to 150 μm. These cavities reveal the rock grains that are lying under the coating. Small mineral fragments are dispersed sparsely on the polymer layer surface.

Figure 14b presents the front surface of sample—a side view. The uniform polymer coating, a few μm thick, is contaminated with mineral particles. The coating separates from the rock surface, which can result from polymer drying and sample splitting.

## 4. Discussion

1. Knowledge of the rheological parameters of base fluids is indispensable in the design of technological treatment. On this basis, fracturing fluids are selected for a specific type of reservoir rock and for the reservoir conditions. They also prove a specific fluid’s potential to transport the proppant. Apparent viscosity was studied for process fluid solutions with the addition of surfactant A, U-2 or Aerosil 300 nanoadditives, and in certain cases, polymer W. Each time, basic rheological parameters were studied 50% and 70% quality foam at 23 °C. The viscosity of the 50% foam with the addition of a foamer and of both nanoadditives did not differ and was approximately 15 cP. The viscosity coefficient of foam (at *Q_f_* = 70%) with U-2 addition was much higher than that with Aerosil additive. After polymer addition to U-2 nanoparticles, the viscosity significantly increased, in particular at 50% foam quality. The viscosity increased from a few cP for the non-foamed fluid to a few dozen cP in the case of foam with nanoadditive and natural polymer; the foaming resulted in a dozen or so times increase in the S.F. viscosity and in its stability, which was confirmed also by the half-time measurement.
2. The permeability coefficient significantly decreased, in particular in the case of cores treated with non-foamed process fluids. Foamed fluids caused a smaller permeability and porosity reduction than non-foamed fluids. The biggest damage to permeability was caused by a non-foamed fluid with the addition of polymer W. The addition of nanoparticles also caused a reduction of permeability, in particular after the application of Aerosil. Instead, the addition of U-2 sol did not result in a significant reduction of the permeability coefficient, especially after the fluid foaming with N_2_. The estimated permeability damage was approximately 20% smaller for foamed fluids as compared with fluids without nitrogen addition.
3. An average height of the cake for non-foamed fluids, determined by the 3D software in the optical microscope, ranged between 1161 and approximately 30 µm. Instead, in the case of cores treated with foamed fracturing fluids, the measured filtration cake was definitely thinner and was from a few dozen to approximately a dozen µm thick. The results of the presented studies show that the foamed fluid based on U-2 nanoparticles with a foamer addition is the least invasive. Only small traces of a filtration cake in the form of an uneven coat were visible on the surface. Its thickness in the case of U-2 application was estimated at approximately 63 µm, while in the case of fluid with Aerosil 300 addition, it was approximately 1161 µm.
4. The SEM analysis allowed the filtration cake thickness and also the polymer presence in the analyzed rock material to be determined. The results of the presented SEM studies show that foamed fluids are the least invasive, forming an irregular flaky coating on the core surfaces, which was consistent with the analysis using the optical microscope and profilometer. Nanoadditives affected the formation of filtration cake on the sample’s surface, especially in the core damaged with a non-foamed fluid with the Aerosil additive. During the non-foamed fluid filtration, the filtration cake created a pretty compact and more even coating. Its thickness ranged from a few to a few dozen μm.
5. Taking into consideration the foam stability, rheology parameters, and the degree of damage, a foamed fracturing fluid based on 0.1% of U-2 with the addition of 4 mL/L of surfactant is the best fluid. The experimental data showed that the stability foam increased when silica (SiO_2_) nanoparticles were added. For fracturing, SiO_2_ nanoparticle-surfactant-stabilized foam is superior to traditional water based fracturing fluids and causes lower core permeability damage than a traditional F.F. It is recommended for use in hydraulic fracturing, particularly for fracturing stimulation in tight and shale gas reservoirs. The obtained results demonstrate that the suitability of the addition of nanoparticles to fracturing fluid for stimulation will improve its performance.

## Figures and Tables

**Table 1:** Rheological parameters of fluids energized with N_2_ with the application of Aerosil nanoadditive, foam quality of 50% and 70%.

S.F. Composition		*Q_f_* [%]	*t* [min]	*n*′ [-]	*K*′ [Pa·s^n^’]	Dynamic Viscosity at A Given *γ* [mPa·s]		
						40 s^−1^	100 s^−1^	170 s^−1^
Water 4 mL/L A, 0.1% Areosil	1a	Non-foamed	13	0.9988	0.000022	2.0	2.3	2.5
			25	0.9989	0.000026	2.1	2.4	2.6
			38	0.9989	0.000027	2.0	2.3	2.4
	1b	50	13	0.5565	0.002466	23.0	15.3	12.1
			25	0.4125	0.00512	28.1	16.4	12.0
			38	0.5116	0.003098	24.5	15.6	12.1
		70	13	0.4479	0.007347	45.9	27.7	20.6
			25	0.5551	0.004069	37.7	25.1	19.8
			38	0.5939	0.003564	38.2	26.3	21.2

**Table 2:** Rheological parameters of fluids energized with N_2_ with the application of U-2 nanoadditive, foam quality of 50% and 70%.

S.F. Composition		*Q_f_* [%]	*t* [min]	*n*′ [-]	*K*′ [Pa·s^n^’]	Dynamic Viscosity at A Given *γ* [mPa·s]		
						40 s^−1^	100 s^−1^	170 s^−1^
Water 4 mL/L A, 0.1% U-2	2a	Non-foamed	13	0.9907	0.0026	2.6	2.5	2.5
			25	0.999	0.0021	2.4	2.5	2.6
			38	0.999	0.0024	2.6	2.6	2.6
	2b	50	13	0.4816	0.003417	24.2	15.0	11.4
			25	0.5403	0.002568	22.6	14.8	11.6
			38	0.642	0.001544	19.7	14.2	11.8
		70	13	0.5923	0.007221	76.8	52.9	42.6
			25	0.6662	0.005006	69.9	51.5	43.1
			38	0.4845	0.011807	84.4	52.6	40

**Table 3:** Rheological parameters of fluids energized with N_2_ with the application of U-2 nanoadditive and natural polymer, foam quality of 50% and 70%.

S.F. Composition		*Q_f_* [%]	*t* [min]	*n*′ [-]	*K*′ [Pa·s^n^’]	Dynamic Viscosity at A Given *γ* [mPa·s]		
						40 s^−1^	100 s^−1^	170 s^−1^
Water 4 mL/L A, 0.1% U-2 0.1% W	3a	Non-foamed	13	0.9989	0.0019	2.9	3.3	3.5
			25	0.9989	0.0017	2.5	2.8	3.0
			38	0.9989	0.0014	2.5	2.8	3.1
	3b	50	13	0.4283	0.006129	35.6	21.1	15.6
			25	0.4123	0.006996	38.3	22.4	16.4
			38	0.4187	0.006726	37.7	22.1	16.3
		70	13	0.7154	0.004226	70.8	54.6	46.9
			25	0.7277	0.004209	73.8	57.5	49.8
			38	0.7496	0.004297	81.7	64.9	56.9

**Table 4:** Measurements of foamed S.F. half-time with addition of 50% and 70% of N_2_.

S.F. Composition	*Q_f_* [%]	Foam Half-Time [s]
4 mL/L A	50	30
4 mL/L A	70	60
0.1% Areosil, 4 mL/L A	50	60
0.1% Areosil, 4 mL/L A	70	90
0.1% U-2, 4 mL/L A	50	80
0.1% U-2, 4 mL/L A	70	240
0.1% U-2, 4 mL/L A, 0,1% W	50	360
0.1% U-2, 4 mL/L A, 0,1% W	70	390

**Table 5:** Results of the porosity ratio and permeability coefficient measurement before and after the performance of damaging tests.

Fluids Injected Through the Core		Core Number	*k*_0_ [md]	*k_f_* [md]	% k_red_	*φ*_0_ [%]	φ_f_ [%]	% φ_red_
S.F. 1a	0.1% Areosil, 4 mL/L A Non-foamed	3231	5.03	1.93	61.00	15.05	13.53	10.09
S.F. 1b	0.1% Areosil, 4 mL/L A Foamed with N_2_	3232	4.72	2.78	41.10	15.20	13.84	8.95
S.F. 2a	0.1% U-2 4 mL/L A Non-foamed	3226	4.11	2.06	49.88	15.70	13.37	14.81
S.F. 2b	0.1% U-2 4 mL/L A Foamed with N_2_	3224	3.96	2.99	24.49	15.07	14.60	3.12
S.F. 3a	0.1% U-2 4 mL/L A 0.1% W Non-foamed	3233	7.65	2.32	69.67	15.80	14.08	8.10
S.F. 3b	0.1% U-2 4 mL/L A 0.1% W Foamed with N_2_	3229	6.92	4.82	30.35	15.77	15.38	2.47

## Abbreviations

The following abbreviations are used in this manuscript:

%*k_red_*	permeability reduction
%*Φ_red_*	porosity reduction
A	anionic foamer
EOR	Enhanced oil recovery
F	Feld feldspar
F.F.	Fracturing fluids
HF	hydraulic fracturing
LHP	lipophobic and hydrophilic nanoparticles
HPG	hydroxypropyl guar
K	consistency factor
*k*_0_	initial core permeability
*k_f_*	final core permeability
n	flow index
Q	quartz
*Q_f_*	foam quality
S.F.	stimulation fluids
*T*	temperature
*t*	test time
TEOS	tetraethoxysilane
W	fast hydrating guar gum (HPG)
*γ*	shear rate
*Φ*_0_	initial core porosity
*Φ*_f_	final core porosity
*k_o_*	initial core permeability
*k_f_*	final core permeability
